# Non-alcoholic fatty liver disease among sasang constitutional types: a population-based study in Korea

**DOI:** 10.1186/s12906-015-0925-8

**Published:** 2015-11-07

**Authors:** Seung Ku Lee, Dae Wui Yoon, Si Woo Lee, Jong Yeol Kim, Jin Kwan Kim, Chol Shin

**Affiliations:** Institute of Human Genomic Study, College of Medicine, Korea University Ansan Hospital, 516 Gojan-1-dong, Danwon-gu, Gyeonggi-do, Ansan, 425-707 Republic of Korea; Constitutional Medicine and Diagnosis Research Group, Korea Institute of Oriental Medicine, 461-24 Jeonmin-dong, Yuseong-gu, Daejeon, 305-811 Republic of Korea; Medical Research Division, Korea Institute of Oriental Medicine, 461-24 Jeonmin-dong, Yuseong-gu, Daejeon, 305-811 Republic of Korea; Department of Biomedical Laboratory Science, Jungwon University, 85 Munmu-ro, Goesan-eup, Goesan-gun, Chungbuk, Republic of Korea; Department of Pulmonary, Sleep and Critical Care Medicine, College of Medicine, Korea University Ansan Hospital, Ansan, Republic of Korea

**Keywords:** Non-alcoholic fatty liver disease, Sasang constitutional types, Liver attenuation index, Computed tomography

## Abstract

**Background:**

Non-alcoholic fatty liver disease (NAFLD) is the most common cause of chronic liver disease and is highly prevalent in populations with metabolic conditions such as obesity and type II diabetes. Specific types of Sasang constitution can act as a risk factor for metabolic diseases, but there are no studies addressing the association between the Sasang constitutional types (SCTs) and NAFLD.

**Methods:**

A total of 1184 individuals (508 males, 676 females) that enrolled in the Korean Genome and Epidemiology Study were included in the present study. Classification of SCTs was done with an integrated diagnostic model. NAFLD was diagnosed when the liver attenuation index (LAI) value was <5 Hounsfield units using computed tomography. Relationships between the SCTs and NAFLD were analyzed using multiple logistic regressions.

**Results:**

The average LAI was 13.3 ± 6.0 in the So-eum (SE) type, 12.3 ± 7.0 in the So-yang (SY) type, and 6.5 ± 9.9 in the Tae-eum (TE) type. Prevalence of NAFLD was 4.7 % in the SE type, 14.0 % in the SY type, and 34 % in the TE type. Even after adjusting for possible confounders, the SY and TE types continued to show a 3.90-fold (95 % CI, 1.60-9.51; *P* = 0.0028) and 3.36-fold (95 % CI, 1.42-7.92; *P* = 0.0057) increase in chance of having NAFLD, respectively, compared with the SE type. In the additional analysis including only non-obese subjects, the odds ratio of NAFLD was 3.27 (95 % CI, 1.29-8.29; *P* = 0.0126) in the SY type and 3.53 (95 % CI, 1.30-9.58; *P* = 0.0134) in the TE type compared with SE type. In the multivariate analysis to determine which parameter had an independent association with NAFLD, higher body mass index, alanine aminotransferase (ALT), triglyceride (TG), and low high-density lipoprotein cholesterol were independently associated with developing NAFLD in the SY type. In contrast, male sex, alcohol consumption, higher ALT, TG, and fasting glucose were risk factors for NAFLD in the TE type.

**Conclusions:**

These results indicated that the SY and TE types are independent risk factors for NAFLD.

## Background

Sasang constitutional medicine (SCM), one of Korea’s traditional medicines, was initiated by Jema Lee (1837–1900) and has a history of more than 100 years of practice [1]. In SCM, people can be classified into four types, the Sasang constitutional types (SCTs), based on the theory that each SCT has specific physiological, physical, and psychological traits, different responses to herbs and sensitivities to pathologic conditions, and different internal organ functional activity [2, 3]. The four groups are: Tae-yang (TY), Tae-eum (TE), So-yang (SY), and So-eum (SE). According to the traditional SCM theory, each SCT represents different temperaments, body shapes, and functional activity of the four viscera - lungs, liver, spleen, and kidneys [2, 3]. It has been suggested that the TY type is progressive, positive, and has a more developed lung and nape of the neck area and a less developed liver area. The TE type has a gentle, commercial, and endurable personality, and has a more developed liver area and a less developed lung area. Typically, the SE type can be characterized by a negative, selfish, and nervous personality, and people who are belonged to this type typically have a more developed kidney and a less developed spleen. The SY type has temperament of unstable and easily gets bored. Unlike the SE type, people who are classified as the SY type had a more developed spleen area and a less developed spleen area. Several studies have also shown that specific SCTs are prone to specific chronic diseases (e.g., hypertension (HTN), diabetes mellitus (DM), and sleep apnea), and to metabolic disturbances [4–7]. The TE type has a higher level of body mass index (BMI), triglycerides (TG), and blood pressure (BP) compared with the SY and SE types [5–7]. Moreover, the TE type is regarded as an independent risk factor for HTN [5], DM [7], and sleep apnea [6]. The TE and SY types have a higher risk for metabolic syndrome (MS) [8], a cluster of metabolic risk factors characterized by high BP, high blood glucose, low high-density lipoprotein (HDL) cholesterol, and abdominal obesity, compared with the SE type.

Non-alcoholic fatty liver disease (NAFLD) is the most common cause of chronic liver disease. It includes a wide spectrum of conditions, such as non-alcoholic steatohepatitis (NASH) with or without fibrosis, cirrhosis, and associated complications [9, 10]. Patients have no or a low daily alcohol consumption (≤ 30 g/day for men, ≤ 20 g/day for women) [11]. The prevalence of NAFLD in the general population varies in different epidemiologic studies. In the US [12] and Europe [11, 13], researchers report a 10-35 % and 20-30 % prevalence of NAFLD, respectively. Similar to the US, the estimated prevalence of NAFLD in the Asia-Pacific region ranges from 5 % to 30 % [14]. In Korea, NAFLD prevalence ranges from 18 % to 22 % [15, 16].

NAFLD has a close relationship with type II DM and features of MS. It was reported that the prevalence of NAFLD in patients with type II DM was 69 % [17]. The prevalence of simple steatosis in the obese ranges from 30 % to 37 % [18, 19]. Moreover, insulin resistance has been shown to be positively correlated with the degree of steatosis [20]. Conversely, patients with steatosis had higher BMIs, insulin resistance scores, waist/hip ratios, and TG [21].

Although specific SCTs are independent risk factors for pathologic conditions such as DM and MS, which are highly associated with NAFLD and account for metabolic disturbances, there is a lack of research about the relationship between the SCTs and NAFLD. The identification of the specific SCTs who are vulnerable to NAFLD may be helpful in predicting susceptibility to the disease, early intervention, and management of high-risk individuals. With this in mind, we investigated the relationship between SCT and NAFLD in a large population-based cohort study.

## Methods

### Subject

The subjects were selected from cohort members who enrolled in the Korean Genome and Epidemiology Study (KoGES). Among 1824 participants who were both followed-up and grouped as an SCT from 2009 to 2011, 1479 individuals who completed computed tomography (CT) scanning were selected for the analysis. There were no significant differences in aspartate aminotransferase (AST) and alanine aminotransferase (ALT) levels in 1824 participants followed-up and in the remaining cohort members who were lost during the period between 2009 and 2011. Then, 295 subjects who had heavy alcohol intake (more than 140 g/week for men, 70 g/week for women), 69 subjects who had a history of viral infection (hepatitis B antigen or hepatitis C antigen positive), and five subjects who suffered a chronic liver disease including hepatocellular carcinoma and cirrhosis, were excluded from the analysis [22]. The remaining 1184 subjects (508 males, 676 females) were ultimately analyzed in the study. Each participant signed written informed consent form before the examination. Subjects underwent physical examinations, laboratory and questionnaire evaluations, and a review of their medical histories. The study protocol was approved by the Institutional Review Board of Korea University Ansan Hospital. Consistent with the previous finding that the TY type is extremely low in the population [7], there were no subjects classified as the TY type in our study. Therefore, only the data from the TE, SE, and SY types was evaluated in the present study.

### Classification of the SCTs

Each constitutional type was classified by an integrated Sasang constitutional diagnostic model developed by Do et al. [23]. Unlike the Questionnaire for Sasang Constitutional Classification II (QSCC II), a commonly used questionnaire focusing on individual qualitative components to determine the SCT [24], this newly developed diagnostic model integrates four individual quantitative components, including facial image, body shape, voice analysis, and questionnaire responses into a single model, increasing the validity of the diagnostic model. The accuracy of the current diagnostic method is higher than that of QSCC II (64 % in males and 55.2 % in females in an integrated diagnostic model vs. 51 % in total with the QSCC II).

Feature variables were acquired from facial images taken with a digital camera and automatically extracted via image processing techniques. As candidate variables to express body shape characteristics, the following parameters were measured or calculated: forehead circumference, neck circumference, axillary circumference, chest circumference, rib circumference, waist circumference, pelvic circumference, hip circumference, height, weight, and body mass index. For voice analysis in the diagnostic model, more than eighty features were extracted by two voice analysis programs, Hidden Markov Model Toolkit and Praat, and processed with a genetic algorithm-based feature selection technique. Each subject also completed a questionnaire composed of 67 multiple-choice questions with binary variables representing personality characteristics and physiologic symptoms.

### Diagnosis of NAFLD

NAFLD was diagnosed using non-invasive computed tomography (CT) scanning. Study procedures for CT are available in a previous report [25]. Briefly, the abdominal adipose tissue area was quantified by a single slice CT scan (Brilliance 64; Philips, Cleveland, OH, USA) and the images were converted into files compatible with a commercial software program (EBW; Philips, Cleveland, OH, USA). The liver attenuation index (LAI), derived from the difference between the mean hepatic and splenic attenuation, was used as a parameter for the diagnosis of NAFLD. A LAI of <5 Hounsfield Units (HU) is a known predictor of >5 % steatosis [26], and histological confirmation of NAFLD requires a minimum of 5 % steatosis [27]. Therefore, NAFLD was defined when the LAI value was <5 HU in this study.

### Anthropometric measurements and definition of variables and disease

BMI was calculated as weight in kilograms divided by the square of the height in meters (kg/m^2^) measured to the nearest 0.1 cm or 0.1 kg. Waist circumference (cm) was measured at the narrowest point between the lower rib and the iliac crest. BP was measured in a sitting position with a mercury sphygmomanometer on the non-dominant arm. We calculated beverage-specific alcohol consumption in g/day on the basis of the alcohol content (4.5 % for beer, 13 % for wine, 40 % for hard liquor, 22 % for soju, 15 % for chungju, and 6 % for makgeolli), the frequency of drinking, and the amount consumed as previously reported [28]. Leisure-time physical activity was evaluated using questionnaires covering the type of activity, frequency, and duration. A metabolic equivalent (MET) score was assigned to each sports activity based on the compendium of physical activities. Time spent per week performing each activity was multiplied by the MET value of the activity to obtain the total MET-minutes per week. MS was defined according to the modified version of a diagnostic criteria proposed by the National Cholesterol Education Program Adult Treatment Panel III [29], as the presence of three or more of the following clinical criteria: central obesity based on Asia–Pacific criteria (waist circumference ≥90 cm for men and ≥80 cm for women), hypertriglyceridemia (TG ≥150 mg/dl), low HDL cholesterol (HDL cholesterol <40 mg/dl for men and <50 mg/dl for women), high BP (systolic/ diastolic pressure ≥130/80 mmHg or use of antihypertensive medication), and high fasting glucose (fasting glucose ≥110 mg/dl or use of antihyperglycemic agents). The homeostasis model of assessment for insulin resistance (HOMA-IR) was calculated as fasting glucose (mmol/L) x fasting insulin (μU/mL)/22.5. All biochemical analyses for TG, fasting plasma glucose, and HDL cholesterol were conducted in the Seoul Clinical Laboratories (Seoul, South Korea). HTN was defined as systolic BP ≥ 140 mmHg, diastolic BP ≥ 90 mmHg, or the use of antihypertensive medication [30]. DM was defined when fasting glucose exceeded 126 mg/dl or antihyperglycemic agents or insulin were used. Obesity was defined as BMI ≥ 27.5 based on the guideline for Asian populations [31].

### Statistical analysis

Data are expressed as a mean ± standard deviation (SD). The significant differences of the means were evaluated using one-way analysis of variance (ANOVA) for continuous variables and chi-square test for categorical variables. Bonferroni’s *post hoc* test was performed for multiple comparisons. In addition, we conducted a multiple logistic regression analysis to estimate an odds ratio (OR) of the presence of NAFLD in relation to different SCTs with a 95 % confidence interval (CI) and *P* value. The potential confounding variables adjusted in the multivariate Model 1 were age, sex, BMI, alcohol consumption, alanine aminotransferase (ALT), and physical activity. Model 2 added the presence of each metabolic component (abdominal obesity, hypertriglyceridemia, low HDL cholesterol, high BP, and high fasting plasma glucose) to Model 1. We also conducted an additional multiple logistic regression to identify an independent association only in the non-obese group. Statistical analysis was performed with SAS version 9.3 (SAS Institute, Cary, North Carolina, USA). All *P* values <0.05 were considered as statistically significant.

## Results

### General characteristics of participants

A total 1184 subjects (508 males and 676 females) who completed CT scanning were included in the analysis. The general characteristics of the participants according to their SCTs are shown in Table 1. The mean age of total subjects was 57.3 ± 7.4 years. The TE type was older, obese, and has a lower proportion of females than the other types. The TE type consumed more alcohol and had higher levels of ALT and HOMA-IR than the other two types. The prevalence of HTN, DM, and NAFLD was the highest in the TE type compared with the SE and SY types. Compared with the SE type, the SY had a significantly higher prevalence of NAFLD. LAI in the TE type was lower than those of the SE and SY types. Values of all metabolic components were significantly higher in the TE type compared with the other types. The TE type had the highest prevalence of MS among the SCTs. Physical activity was not statistically different among the SCTs.

**Table 1:** General characteristics according to Sasang constitution type

Variable	SE	SY	TE	*P* value
No. of cases	172	407	605	
Age (yr)	55.3 ± 6.0^a**^	56.0 ± 6.4^**^	58.8 ± 7.9	<.0001
Female, *n* (%)	94 (54.7)^*****^	285 (70.0)^**^	297 (49.1)	<.0001
BMI (kg/m^2^)	21.3 ± 1.6^**,******^	22.9 ± 2.0^**^	26.6 ± 2.4	<.0001
Alcohol consumption (g/day)	1.8 ± 4.0^*^	1.8 ± 3.9^**^	3.1 ± 5.3	<.0001
ALT (IU/L)	23.1 ± 24.1^*^	21.8 ± 10.0^**^	27.3 ± 16.2	<.0001
HOMA-IR^b^	1.5 ± 0.6^**^	1.7 ± 0.8^**^	2.6 ± 3.5	<.0001
Physical activity^c^	159.5 ± 195.5	189.7 ± 276.4	189.0 ± 258.2	0.37
LAI (HU)	13.3 ± 6.0^**^	12.3 ± 7.0^**^	6.5 ± 9.9	<.0001
NAFLD [LAI < 5, *n* (%)]	8 (4.7)^**,****^	57 (14.0)^**^	206 (34.0)	<.0001
HTN, *n* (%)	35 (20.3)^**^	99 (24.3)^**^	266 (44.0)	<.0001
DM, n (%)	13 (7.6)^**^	50 (12.3)^**^	164 (27.1)	<.0001
Metabolic components^d^				
Abdominal obesity, *n* (%)	5 (2.9)^**,***^	35 (8.6)^**^	293 (48.5)	<.0001
Hypertriglyceridemia, *n* (%)	28 (16.3)^**,***^	100 (24.6)^**^	248 (41.0)	<.0001
Low HDL cholesterol, *n* (%)	122 (70.9)^**,****^	232 (57.0)^**^	293 (48.4)	<.0001
High BP, *n* (%)	45 (26.2)^**^	124 (30.6)^**^	317 (52.7)	<.0001
High FPG, *n* (%)	8 (4.7)^**,***^	40 (9.8)^**^	140 (23.1)	<.0001
MS, *n* (%)^e^	6 (3.5)^**,****^	41 (10.1)^**^	233 (38.5)	<.0001

*Abbreviations: TE* Tae-eum type, *SE* So-eum type, *SY* So-yang type, *BMI* body mass index, *ALT* alanine aminotransferase, *HOMA-IR* homeostasis model of assessment for insulin resistance, *MET* metabolic equivalents, *LAI* liver attenuation index, *HU* Hounsfield units, *NAFLD* non-alcoholic fatty liver disease, *HTN* hypertension, *DM* diabetes mellitus, *HDL* high density lipoprotein, *BP* blood pressure, *FPG* fasting plasma glucose, *MS* metabolic syndrome

^a^Values are mean ± SD

^b^Calculated as fasting glucose (mmol/L) x fasting insulin (μU/mL)/22.5

^c^Total metabolic equivalents per week (minutes)

^d^Defined as waist circumference ≥90 cm for men and ≥80 cm for women for abdominal obesity; TG ≥150 mg/dl for hypertriglyceridemia; HDL cholesterol <40 mg/dl for men and <50 mg/dl for women for low HDL cholesterol; systolic/ diastolic pressure ≥130/85 mmHg for high BP; fasting plasma glucose ≥110 mg/dl for high FPG

^e^Presence of 3 or more of metabolic components

^*^ *P* <0.01 vs. TE type

^**^ *P* <0.0001 vs. TE type

^***^ *P* <0.05 vs. SY type

^****^ *P* <0.01 vs. SY type

^*****^ *P* <0.001 vs. SY type

^******^ *P* <0.0001 vs. SY type

### Odds ratios (ORs) for NAFLD according to the SCTs

In order to estimate ORs for NAFLD in relation to constitutional types, a logistic regression analysis was conducted (Table 2). Under crude analysis, the SY and TE types were more likely to have NAFLD compared with the SE type. In the analysis adjusted for age, sex, BMI, alcohol consumption, ALT, and physical activity (Model 1), the SY and TE types had 4.21 times (95 % CI, 1.75-10.13; *P* = 0.0028) and 3.31 times (95 % CI: 1.43-7.67, *P* = 0.0051) higher odds of having NAFLD than that of the SE type, respectively. In the analysis additionally adjusted for all metabolic components (Model 2), the SY and TE types continued to show a 3.90-fold (95 % CI, 1.60-9.51; *P* = 0.0028) and a 3.36-fold (95 % CI, 1.42-7.92, *P* = 0.0057) greater chance of having NAFLD than the SE type, respectively.

**Table 2:** Odds ratio of NAFLD in relation to Sasang constitution type

	Estimated odds ratios of NAFLD (95 % CI)		
	SE	SY	TE
No. of normal cases (%)	164 (95.4 %)	349 (86.0 %)	399 (66)
No. of NAFLD cases (%)	8 (4.6)	58 (14.0)	206 (34)
Crude	reference	10.58 (5.10–21.94), *P* < .0001	3.34 (1.56–7.16), *P* = 0.002
Model 1^a^	reference	4.21 (1.75–10.13), *P* = 0.0013	3.31 (1.43–7.67), *P* = 0.0051
Model 2^b^	reference	3.90 (1.60–9.51), *P* = 0.0028	3.36 (1.42–7.92), *P* = 0.0057

*Abbreviations: TE* Tae-eum type, *SE* So-eum type, *SY* So-yang type, *NAFLD* non-alcoholic fatty liver disease

^a^Model 1: adjusted for age, sex, BMI, alcohol consumption, ALT, and physical activity

^b^Model 2: adjusted for Model 1 + metabolic components

### Odds ratios (ORs) for NAFLD in the non-obese subjects according to the SCTs

To evaluate the association between NAFLD and the SCTs in non-obese subjects, we excluded subjects having BMIs ≥ 27.5 (n = 190), and the remaining 994 participants were used in the analysis (Table 3). In the multivariate analysis (Model 2) for the association between NAFLD and the SCTs, the ORs of NAFLD were 3.27-fold (95 % CI, 1.29-8.29, *P* = 0.0126) for the SY type, and 3.53-fold (95 % CI, 1.30-9.58; *P* = 0.0134) for the TE type, greater than the SE type.

**Table 3:** Odds ratio of NAFLD in relation to Sasang constitution type in non-obese subjects

	Estimated odds ratios of NAFLD (95 % CI)		
	SE	SY	TE
No. of normal cases (%)	164 (95.9)	345 (86.3)	297 (70.2)
No. of NAFLD cases (%)	7 (4.1)	55 (13.8)	126 (29.8)
Crude	reference	3.74 (1.66–8.38), *P* = 0.0014	9.94 (4.54–21.78), *P* < .0001
Model 1^a^	reference	3.30 (1.32–8.22), *P* = 0.0104	3.70 (1.37–9.99), *P* = 0.0099
Model 2^b^	reference	3.27 (1.29–8.29), *P* = 0.0126	3.53 (1.30–9.58), *P* = 0.0134

*Abbreviations: TE* Tae-eum type, *SE* So-eum type, *SY* So-yang type, *NAFLD* non-alcoholic fatty liver disease

^a^Model 1: adjusted for age, sex, BMI, alcohol consumption, ALT, and physical activity

^b^Model 2: adjusted for Model 1 + metabolic components

Non-obese was defined as BMI <27.5

### Logistic regression analysis of possible risk factors for NAFLD in the SY and TE types

We conducted an additional multivariate logistic regression analysis with possible risk factors for NAFLD to determine which parameters had an independent association with NAFLD in the SY and TE types. In the SY type, higher BMI, ALT, TG, and low HDL cholesterol were independently associated with developing NAFLD. In contrast, male sex, alcohol consumption, higher ALT, TG, and fasting glucose were the risk factors for NAFLD in the TE type. Only two common factors, higher ALT and TG, were seen in both the SY and the TE types.

## Discussion

The present cohort study found that the SY and TE types are associated with an increased risk of NAFLD, even after taking into account possible confounders, independent of obesity. We also found that the risk factors for NAFLD are different in the SY and TE types.

The interesting finding was that, although the SY type had a lower value or prevalence of risk factors for NAFLD than the TE type, the risk of having NAFLD was higher in the SY type than the TE type. The reason why the ORs for NAFLD is different is unknown, but, as revealed by logistic regression analysis of possible risk factors for NAFLD in Table 4, it may be due to the difference in the sensitivity to each risk factor between the SY and TE types. For example, high BMI, TG, and low HDL cholesterol were significant risk factors for NAFLD in the SY type, but they were not in the TE type. On the other hand, male sex, alcohol consumption, and high fasting glucose were the risk factors only in the TE type. Further studies are required to clarify what causes this difference among the SCTs.

**Table 4:** Logistic regression analysis of possible risk factors for NAFLD in the SY and TE types

	SY type			TE type		
Variable	OR	95 % CI	*P* value	OR	95 % CI	*P* value
Age (yr)	1.03	0.97–1.08	0.3568	0.99	0.97–1.02	0.5492
BMI (kg/m^2^)	1.33	1.09–1.62	0.0045	1.08	0.98–1.19	0.132
Sex (female)	0.51	0.23–1.13	0.0976	0.32	0.19–0.52	<.0001
Alcohol consumption (g/day)	1.02	0.94–1.11	0.6112	0.91	0.87–0.95	<.0001
ALT (IU/L)	1.05	1.02–1.08	0.0015	1.06	1.04–1.07	<.0001
Abdominal obesity	0.53	0.15–1.83	0.3146	1.66	0.98–2.82	0.0584
Hypertriglyceridemia	3.25	1.64–6.43	0.0007	1.59	1.07–2.37	0.0225
Low HDL cholesterol	0.37	0.14–0.76	0.0075	1.13	0.75–1.69	0.5648
High BP	1.85	0.91–3.76	0.0906	1.48	0.97–2.27	0.0719
High FPG	2.11	0.85–5.24	0.1092	1.77	1.12–2.80	0.0144

*Abbreviations: TE* Tae-eum type, *SY* So-yang type, *NAFLD* non-alcoholic fatty liver disease, *BMI* body mass index, *ALT* alanine aminotransferase, *HDL* high density lipoprotein, *BP* blood pressure, *FPG* fasting plasma glucose

NAFLD was defined as LAI <5

Because NAFLD is strongly associated with obesity [12, 32] and our SCTs had a significantly different degree of obesity, we repeated the analysis to examine the association between NAFLD and the SCTs in non-obese subjects having BMI <27.5 (Table 3). Similar to the results of the multivariate analysis including all subjects (Table 2), the SY and TE types continued to show a significantly higher risk of having NAFLD compared with the SE type, implying that obesity is not a major risk factor for NAFLD in the SY and TE types. The ORs, however, was higher in the TE type than in the SY type, inconsistent with the results of Table 2. Obesity is a cause of NAFLD’s increasing prevalence in western countries [33, 34], but obesity as a risk factor for NAFLD may not be generalizable to Asian populations. In cohort studies, a high prevalence of NAFLD was reported in the Asians who had significantly lower BMI [35, 36]. Although not entirely elucidated, one possible explanation for the discrepancy between obesity and NAFLD is the ethnic differences in the distribution of body fat, given that Asians have a more central adiposity and visceral fat deposition [37–40].

The reason why the SY and TE type have higher risk of having NAFLD than the SE type is unknown, but several speculations could be made. First, dysfunctions of lipid metabolism pathway caused by genetic variations can exist in those types. It has been previously reported that the TE type is associated with genetic variants of pathways which have previously been reported to cause HTN [41, 42]. However, there have been no studies that investigated the association between specific genetic polymorphisms related with dysfunctions of lipid metabolism and SCTs, thus further studies are required. Second, sleep apnea also may contribute to the NAFLD in TE and SY types. We previously reported that TE and SE type had a 2.3 and 1.9 greater risk of having obstructive sleep apnea (OSA), respectively, compared to the SE type [6]. Given that OSA is associated with NAFLD [43, 44], the presence of OSA in these types could partially explain why the types had greater risk for NAFLD compared to the SE type.

The main strength of our study includes its randomly-sampled population-based study design and the large sample size. There are some limitations worth noting in this study. First, we identified NAFLD by CT scanning, not by liver biopsy, which is the diagnostic gold standard for NAFLD [45]. Additionally, the identification of chronic liver disease, to exclude it from the analysis, was done based on a questionnaire, increasing the chance of underestimated NAFLD prevalence. But it is not possible to perform liver biopsies in an epidemiological study because it is an invasive, dangerous, and time-consuming procedure. CT is a good alternative method because it is non-invasive and performs well in the diagnosis of greater degrees of steatosis (>30 %) with a sensitivity of 82 % and a specificity of 100 % [25]. Thus, it is more suitable for diagnosis of NAFLD than liver biopsy in the cohort study. Second, the age distribution of the study participants was not even, but biased toward middle age. The prevalence of NAFLD increases with age, and therefore, the present findings cannot be generalized to young age groups. Third, accuracy of classification method used in this study is low (64 % in males and 55.2 % in females). Fourth, dietary information, including amount of fat intake, was not examined in the present study.

## Conclusions

The SY and TE types are independent risk factors for NAFLD. These findings may contribute to the early prevention and management of high-risk individuals of NAFLD according to the SCTs, and ultimately, to personalized medicine. Further investigation is needed to confirm the risk factors for NAFLD according to the SCTs and to explore the physiological mechanisms underlying the association.

## References

[1] Shim EB, Lee S, Kim JY, Earm YE (2008). Physiome and Sasang Constitutional Medicine. J Physiol Sci.

[2] Chae H, Lyoo IK, Lee SJ, Cho S, Bae H, Hong M (2003). An alternative way to individualized medicine: psychological and physical traits of Sasang typology. J Altern Complement Med.

[3] Kim JY, Pham DD (2009). Sasang constitutional medicine as a holistic tailored medicine. Evid Based Complement Alternat Med.

[4] Jang E, Baek Y, Park K, Lee S (2013). The sasang constitution as an independent risk factor for metabolic syndrome: propensity matching analysis. Evid Based Complement Alternat Med.

[5] Lee J, Lee E, Yoo J, Kim Y, Koh B (2011). The Sasang constitutional types can act as a risk factor for hypertension. Clin Exp Hypertens.

[6] Lee SK, Yoon DW, Yi H, Lee SW, Kim JY, Shin C (2013). Tae-eum type as an independent risk factor for obstructive sleep apnea. Evid Based Complement Alternat Med.

[7] Lee TG, Koh B, Lee S (2009). Sasang constitution as a risk factor for diabetes mellitus: a cross-sectional study. Evid Based Complement Alternat Med.

[8] Song KH, Yu SG, Kim JY (2012). Prevalence of Metabolic Syndrome according to Sasang Constitutional Medicine in Korean Subjects. Evid Based Complement Alternat Med.

[9] Matteoni CA, Younossi ZM, Gramlich T, Boparai N, Liu YC, McCullough AJ (1999). Nonalcoholic fatty liver disease: a spectrum of clinical and pathological severity. Gastroenterology.

[10] Younossi ZM, Stepanova M, Rafiq N, Makhlouf H, Younoszai Z, Agrawal R (2011). Pathologic criteria for nonalcoholic steatohepatitis: interprotocol agreement and ability to predict liver-related mortality. Hepatology.

[11] Scaglioni F, Ciccia S, Marino M, Bedogni G, Bellentani S (2011). ASH and NASH. Dig Dis.

[12] Vernon G, Baranova A, Younossi ZM (2011). Systematic review: the epidemiology and natural history of non-alcoholic fatty liver disease and non-alcoholic steatohepatitis in adults. Aliment Pharmacol Ther.

[13] Bedogni G, Miglioli L, Masutti F, Tiribelli C, Marchesini G, Bellentani S (2005). Prevalence of and risk factors for nonalcoholic fatty liver disease: the Dionysos nutrition and liver study. Hepatology.

[14] Amarapurkar DN, Hashimoto E, Lesmana LA, Sollano JD, Chen PJ, Goh KL (2007). How common is non-alcoholic fatty liver disease in the Asia-Pacific region and are there local differences?. J Gastroenterol Hepatol.

[15] Kim NH, Park J, Kim SH, Kim DH, Cho GY, Baik I (2014). Non-alcoholic fatty liver disease, metabolic syndrome and subclinical cardiovascular changes in the general population. Heart.

[16] Park SH, Jeon WK, Kim SH, Kim HJ, Park DI, Cho YK (2006). Prevalence and risk factors of non-alcoholic fatty liver disease among Korean adults. J Gastroenterol Hepatol.

[17] Leite NC, Salles GF, Araujo AL, Villela-Nogueira CA, Cardoso CR (2009). Prevalence and associated factors of non-alcoholic fatty liver disease in patients with type-2 diabetes mellitus. Liver Int.

[18] Abrams GA, Kunde SS, Lazenby AJ, Clements RH (2004). Portal fibrosis and hepatic steatosis in morbidly obese subjects: A spectrum of nonalcoholic fatty liver disease. Hepatology.

[19] Boza C, Riquelme A, Ibanez L, Duarte I, Norero E, Viviani P (2005). Predictors of nonalcoholic steatohepatitis (NASH) in obese patients undergoing gastric bypass. Obes Surg.

[20] Angelico F, Del Ben M, Conti R, Francioso S, Feole K, Fiorello S (2005). Insulin resistance, the metabolic syndrome, and nonalcoholic fatty liver disease. J Clin Endocrinol Metab.

[21] Targher G, Bertolini L, Scala L, Poli F, Zenari L, Falezza G (2004). Decreased plasma adiponectin concentrations are closely associated with nonalcoholic hepatic steatosis in obese individuals. Clin Endocrinol (Oxf).

[22] Farrell GC, Larter CZ (2006). Nonalcoholic fatty liver disease: from steatosis to cirrhosis. Hepatology.

[23] Do JH, Jang E, Ku B, Jang JS, Kim H, Kim JY (2012). Development of an integrated Sasang constitution diagnosis method using face, body shape, voice, and questionnaire information. BMC Complement Altern Med.

[24] Yoo JH, Kim JW, Kim KK, Kim JY, Koh BH, Lee EJ (2007). Sasangin diagnosis questionnaire: test of reliability. J Altern Complement Med.

[25] Park SH, Kim PN, Kim KW, Lee SW, Yoon SE, Park SW (2006). Macrovesicular hepatic steatosis in living liver donors: use of CT for quantitative and qualitative assessment. Radiology.

[26] Limanond P, Raman SS, Lassman C, Sayre J, Ghobrial RM, Busuttil RW (2004). Macrovesicular hepatic steatosis in living related liver donors: correlation between CT and histologic findings. Radiology.

[27] Hubscher SG (2006). Histological assessment of non-alcoholic fatty liver disease. Histopathology.

[28] Baik I, Shin C (2008). Prospective study of alcohol consumption and metabolic syndrome. Am J Clin Nutr.

[29] Executive Summary of The Third Report of The National Cholesterol Education Program (NCEP) (2001). Expert Panel on Detection, Evaluation, And Treatment of High Blood Cholesterol In Adults (Adult Treatment Panel III). JAMA.

[30] Chobanian AV, Bakris GL, Black HR, Cushman WC, Green LA, Izzo JL (2003). The Seventh Report of the Joint National Committee on Prevention, Detection, Evaluation, and Treatment of High Blood Pressure: the JNC 7 report. JAMA.

[31] WHO Expert Consultation (2004). Appropriate body-mass index for Asian populations and its implications for policy and intervention strategies. Lancet.

[32] Marchesini G, Marzocchi R, Agostini F, Bugianesi E (2005). Nonalcoholic fatty liver disease and the metabolic syndrome. Curr Opin Lipidol.

[33] Charlton MR, Burns JM, Pedersen RA, Watt KD, Heimbach JK, Dierkhising RA (2011). Frequency and outcomes of liver transplantation for nonalcoholic steatohepatitis in the United States. Gastroenterology.

[34] Wong RJ, Ahmed A (2014). Obesity and non-alcoholic fatty liver disease: Disparate associations among Asian populations. World J Hepatol.

[35] Das K, Mukherjee PS, Ghosh A, Ghosh S, Mridha AR, Dhibar T (2010). Nonobese population in a developing country has a high prevalence of nonalcoholic fatty liver and significant liver disease. Hepatology.

[36] Farrell GC, Wong VW, Chitturi S (2013). NAFLD in Asia--as common and important as in the West. Nat Rev Gastroenterol Hepatol.

[37] Aloia JF, Vaswani A, Mikhail M, Flaster ER (1999). Body composition by dual-energy X-ray absorptiometry in black compared with white women. Osteoporos Int.

[38] Du T, Sun X, Yin P, Huo R, Ni C, Yu X (2013). Increasing trends in central obesity among Chinese adults with normal body mass index, 1993–2009. BMC Public Health.

[39] Flegal KM, Shepherd JA, Looker AC, Graubard BI, Borrud LG, Ogden CL (2009). Comparisons of percentage body fat, body mass index, waist circumference, and waist-stature ratio in adults. Am J Clin Nutr.

[40] Rahman M, Temple JR, Breitkopf CR, Berenson AB (2009). Racial differences in body fat distribution among reproductive-aged women. Metabolism.

[41] Kim BY, Jin HJ, Kim JY (2012). Genome-wide association analysis of Sasang constitution in the Korean population. J Altern Complement Med.

[42] Kim BY, Yu SG, Kim JY, Song KH (2012). Pathways involved in sasang constitution from genome-wide analysis in a Korean population. J Altern Complement Med.

[43] Sookojan S, Pirola CJ (2013). Obstructive sleep apnea is associated with fatty liver and abnormal liver enzymes: a meta-analysis. Obes Surg.

[44] Türkay C, Ozol D, Kasapoğlu B, Kirbas I, Yıldırım Z, Yiğitoğlu R (2012). Influence of obstructive sleep apnea on fatty liver disease: role of chronic intermittent hypoxia. Respir Care.

[45] Adams LA, Feldstein AE (2010). Nonalcoholic steatohepatitis: risk factors and diagnosis. Expert Rev Gastroenterol Hepatol.

